# Early-life gut microbiome development and its potential long-term impact on health outcomes

**DOI:** 10.20517/mrr.2024.78

**Published:** 2025-04-17

**Authors:** Alejandro Borrego-Ruiz, Juan J. Borrego

**Affiliations:** ^1^Department of Social and Organizational Psychology, National University of Distance Education (UNED), Madrid 28040, Spain.; ^2^Department of Microbiology, University of Málaga, Málaga 29071, Spain.

**Keywords:** Gut microbiome, dysbiosis, infancy, long-term physiological outcomes, early-life stress, mental health

## Abstract

The initial gut colonization of the infant plays a pivotal role in shaping the immune system, developing the intestinal tract, and influencing host metabolism, all of which are strongly influenced by several determinants, such as gestational age at birth, mode of delivery, neonatal feeding practices, early-life stress (ELS), and exposure to perinatal antibiotics. However, resulting gut microbiome (GM) dysbiosis may alter this developmental programming, leading to long-term adverse health outcomes. This narrative review synthesizes current knowledge on early-life GM development and its long-term impact on health. Specifically, it addresses how early-life GM dysbiosis may affect the trajectory of physiological processes, predisposing individuals to conditions such as allergic diseases, metabolic disorders, type 1 diabetes, inflammatory bowel disorders, and atherosclerotic cardiovascular diseases. In addition, it examines the influence of probiotic and prebiotic supplementation during pregnancy and early life in shaping infant GM composition, as well as the impact of ELS-induced GM dysbiosis on mental health. Recent research suggests that the early-life microbiota initiates long-lasting effects, and inadequate or insufficient microbial exposure triggers inflammatory responses associated with several physiological conditions. Although several studies have reported a connection between ELS and the GM during both prenatal and postnatal periods, a unified microbiome signature linked to either prenatal or postnatal stress remains to be fully elucidated. Thus, future studies are needed to establish causality and determine whether modifiable factors affecting the GM could be targeted to improve gut health, especially in children exposed to contextual stress or adverse conditions.

## INTRODUCTION

The colonization of the infant gut by microbes during the perinatal period is essential for the future health of the child, as the interaction between the microbiota and the host plays a key role in the proper development of homeostatic systems^[1]^. Therefore, early colonization has a profound impact on subsequent health and represents a window of opportunity for modulating the microbiota toward a healthy composition, potentially leading to long-term beneficial outcomes^[2]^.

The initial gut colonization of the infant is strongly influenced by several determinants, such as gestational age at birth, mode of delivery, neonatal feeding practices, early-life stress (ELS), and exposure to perinatal antibiotics^[3-6]^. The gut microbiome (GM) is established after birth and evolves throughout the lifespan of the host, from infancy to advanced age^[7]^. The GM composition ultimately achieves homeostasis, establishing complex ecological and trophic interrelationships between its microbial members and the human host^[8]^. However, diverse factors can disrupt the microbial balance of the GM, causing a state of dysbiosis^[9]^.

Several clinical and preclinical studies have suggested that GM dysbiosis during the perinatal period may play a pivotal role in the onset of various physiological and neurodevelopmental disorders^[10-12]^. Consequently, disruptions in the GM during critical developmental stages may have persistent effects on health, underscoring the need for early interventions to mitigate the risk of chronic conditions and a deeper understanding of the role of the GM in both physical and mental well-being. In order to provide a comprehensive overview of the topic, this narrative review synthesizes current knowledge on early-life GM development and its long-term impact on health outcomes. Specifically, it addresses how early-life GM dysbiosis may affect the trajectory of physiological processes, predisposing individuals to conditions such as allergic diseases, metabolic disorders, type 1 diabetes (T1D), inflammatory bowel disorders (IBDs), and atherosclerotic cardiovascular diseases (ACVDs). In addition, it examines the influence of probiotic and prebiotic supplementation during pregnancy and early life in shaping infant GM composition, as well as the impact of ELS-induced GM dysbiosis on mental health, with a particular focus on depression.

## EARLY LIFE GM DEVELOPMENT

Until the beginning of the 21st century, the neonatal gut was thought to be a sterile ecosystem (the “sterile womb paradigm”)^[13]^, with microbial colonization believed to commence at birth^[10]^. However, recent findings have challenged this notion, revealing the presence of bacterial cells or DNA in the meconium, placenta, and umbilical cord blood from healthy newborns delivered via cesarean section^[14,15]^. Diverse researchers have postulated the “in utero colonization hypothesis”^[13,16]^ on the basis that probiotics consumed by expectant mothers were identified in both the placenta and in the meconium of term infants^[17,18]^. Perez-Muñoz *et al.* analyzed the evidence supporting these two opposing hypotheses based on (i) the physiological, immunological, and anatomical features of the placenta and fetus; (ii) the methodological approaches currently used to explore microbial populations in the intrauterine environment; (iii) the composition of the fecal microbiome during the first days of life; and (iv) the generation of axenic animals and humans^[13]^. From this analysis, these authors suggested that the “in utero colonization hypothesis” relies on methodologically weak data, likely due to the existence of kitomes. In addition, the consistent success in generating axenic animals via cesarean section provides strong evidence for the sterility of the fetal environment in mammals.

During pregnancy, the mother undergoes several endocrine, immunologic, and metabolic changes aimed at creating an appropriate intrauterine environment for optimal fetal development^[19]^. These modifications promote a pro-inflammatory state, resulting in shifts in the maternal vaginal, intestinal, cutaneous, and oral microbiomes^[20,21]^. Interestingly, the maternal transfer of bacteria to the fetus plays a significant role in the establishment of a healthy neonatal microbiome, which may subsequently influence both the immune system maturation^[22]^ and the neurodevelopment^[23]^. The maternal gut microbiota during late pregnancy is decreased in microbial α-diversity compared to the first trimester, with a decline in the abundance of members of Bacillota but an elevation in the bacteria belonging to the phyla Pseudomonadota and Actinomycetota, as well as to the *Streptococcus* genus^[24,25]^. Furthermore, the administration of probiotics, prebiotics, or synbiotics to the mother during pregnancy has a significant influence on fetal and neonatal GM^[17,26]^. Table 1 shows studies conducted on the effects of probiotic and prebiotic supplementation during pregnancy and early life, and their influence on the composition and diversity of the infant’s GM^[27-47]^.

**Table 1:** Influence of probiotic and prebiotic supplementation during pregnancy and early life on GM composition and diversity in infants

**Ref.**	**Probiotics and prebiotics**	**Effect of probiotic and prebiotic supplementation on infant GM**
Rinne *et al.*^[27]^	*L. rhamnosus* strain GG	At six months, counts of *Bifidobacterium* and *Lactobacillus*/*Enterococcus* were greater in breastfed infants compared to those fed formula. At twelve months, infants who were breastfed for three months and supplemented with probiotics exhibited higher levels of IgM, IgA, and IgG-secreting cells compared to those who received placebo
Gueimonde *et al.*^[28]^	*L. rhamnosus* strain GG	At five days of age, infants whose mothers were supplemented with probiotics had significantly higher colonization by *B. breve* compared to control, but this effect did not persist at three weeks. In addition, maternal supplementation with *L. rhamnosus* did not substantially enhance the gut bifidobacterial diversity in infants at three weeks
Rinne *et al.*^[29]^	*L. rhamnosus* strain GG	Probiotic supplementation during the early months of life did not substantially affect the long-term composition of the gut microbiota, with bifidobacteria remaining the predominant microbiota. At six months, the feces of the placebo group contained a higher abundance of clostridia compared to the probiotic group. After two years of follow-up, the probiotic group showed a lower presence of lactobacilli/enterococci and clostridia than the placebo group, highlighting the role of clostridia as an indicator of microbiota progression in healthy infants
Grönlund *et al.*^[30]^	*B. adolescentis* and *B. bifidum*	Only infants born to allergic, atopic mothers were colonized with *B. adolescentis*. The predominant species found in breast milk was *B. longum*. Allergic mothers had notably lower levels of bifidobacteria in their breast milk compared to non-allergic mothers, and their infants also displayed reduced bifidobacteria counts in their feces
Kukkonen *et al.*^[31]^	Probiotic cocktail: *L. rhamnosus* strain GG, *L. rhamnosus* strain LC705, *B. breve* strain Bb99 and *Propionibacterium freudenreichii* subsp. *shermanii* strain JS Prebiotic: GOS	At three and six months, infants in the probiotic group exhibited a significantly higher frequency of colonization by lactobacilli and *Propionibacterium*. Fecal counts of total bifidobacteria and lactobacilli were notably higher at six months. No significant differences were found between the groups regarding fecal bacterial colonization at two years of age. Probiotic treatment presented a negative correlation between the occurrence of atopic diseases and gut colonization by probiotics
Abrahamsson *et al.*^[32]^	*L. reuteri*	At five days of age, the prevalence of *L. reuteri* was notably higher in the probiotic group compared to the placebo group. Despite ongoing supplementation, the prevalence of the probiotic decreased over time in the infants
Niers *et al.*^[33]^	A mixture of probiotic bacteria (*B. bifidum*, *B. animalis* subsp. *lactis*, and *L. lactis*)	The administration of the probiotic bacterial cocktail demonstrated a preventive effect on eczema incidence in high-risk children, with this effect lasting throughout the first two years of life. The intervention group showed significantly higher colonization rates and greater numbers of *L. lactis*
Grönlund *et al.*^[34]^	Combination 1: *L. rhamnosus* + *B. longum* Combination 2: *Lacticaseibacillus paracasei* + *B. longum*	*Bifidobacterium* genus levels at one month and *B. longum* levels at six months were correlated between mothers and their infants. By six months, the probiotic intervention significantly influenced the mother-infant relationship in fecal bifidobacterial counts, although no notable effects were observed on colonization frequencies, diversity, and similarity indices. Maternal colonization with *B. bifidum* had the most consistent impact on the infant’s bifidobacterial microbiota
Grześkowiak *et al.*^[35]^	Combination 1: *L. rhamnosus* strain LPR + *B. longum* strain BL999 Combination 2: *L. paracasei* strain ST11 + *B. longum* strain BL999	At the genus level, *Bifidobacterium* counts varied significantly across the study groups, with the lowest counts observed in the combination 1 group compared to the placebo. However, the relative abundance of *Bifidobacterium* did not show significant differences between the groups. Infants whose mothers received combination 1 probiotics exhibited a notable difference in the relative abundance of the *Lactobacillus*-*Enterococcus* group compared to the placebo group. No other significant differences were observed between the probiotic and placebo groups in the relative abundance of key bacterial groups, including *Prevotella*, *Clostridium histolyticum*, and *Akkermansia muciniphila*
Ismail *et al.*^[36]^	*L. rhamnosus* strain GG	Prenatal supplementation with *L. rhamnosus* did not significantly influence the fecal microbial diversity in infants at seven days of age
Pärtty *et al.*^[37]^	Probiotic: *L. rhamnosus* strain GG. Prebiotics: Mixture of GOS and polydextrose	The placebo group exhibited a higher percentage of *Clostridium histolyticum* in their stools compared to the probiotic group. Additionally, the ratio of *Lactobacillus*-*Lactococcus*-*Enterococcus* to the total bacterial count was greater in excessive criers than in contented infants at one month of age. The species composition of *Bifidobacterium* also varied between the groups, with *B. longum* subsp. *infantis* being less abundant in the stools of excessive criers compared to contented infants
Enomoto *et al.*^[38]^	*B. longum* strain BB536 and *B. breve* strain M-16 V	At four months of age, infants in the probiotic group showed a significantly higher relative abundance of members of the phylum Bacteroidota compared to the control. No significant differences were obtained in stool samples collected at ten months of age
Bisanz *et al.*^[39]^	*L. rhamnosus* strain GR-1	Infants between 10 and 25 days of age whose mothers were supplemented with probiotics exhibited a threefold increase in the relative abundance of *Bifidobacterium* and a reduction in *Enterobacteriaceae* compared to the control group
Rutten *et al.*^[40]^	*B. bifidum*, *B. animalis* subsp. *lactis* and *L. lactis*	During the supplementation period, the probiotic group showed a higher abundance and prevalence of probiotic species, but this difference diminished once supplementation ceased. At one month of age, bifidobacteria were significantly more abundant in the probiotic group, while *L. lactis* was significantly higher at both two weeks and one month. *L. lactis* was not detected in the placebo group during the intervention and was significantly more abundant at two years in the probiotic group
Avershina *et al.*^[41]^	*L. rhamnosus* strain GG	Infants in the probiotic group exhibited a higher relative abundance of *L. rhamnosus* at ten days and three months of age compared to the control group. However, this difference was not sustained at twelve months or two years. No significant differences were observed between the probiotic and placebo groups in terms of α- or β-diversity of the total microbiota in infant stool samples at three months or two years of age
Korpela *et al.*^[42]^	Probiotics: *B. breve*, *P. freundenreichii* subsp. *shermanii* strain JS, and *L. rhamnosus* strain GG Prebiotic: GOS	The probiotic supplementation had a significant overall impact on microbiota composition, but the effect was influenced by the diet of the infant. In breastfed infants, those receiving probiotics showed a higher relative abundance of lactobacilli and bifidobacteria compared to controls. However, other taxa (clostridia and Gammaproteobacteria) were less abundant in the probiotic group. In formula-fed infants, there was a slight but significant decrease in total bifidobacteria in those supplemented with probiotics. In addition, the genera *Anaerostipes*, *Klebsiella*, and *Veillonella* were more abundant in the formula-fed probiotic group compared to the formula-fed control group
Pärnänen *et al.*^[43]^	Combination 1: *L. rhamnosus* strain LPR and *B. longum* Combination 2: *L. paracasei* and *B. longum*	No significant differences were observed in the abundance of antibiotic resistance genes between the probiotic and placebo groups in the infants
Plummer *et al.*^[44]^	*B. longum* subsp. *infantis* strain BB-02, *Streptococcus thermophilus* strain TH-4 and *B. animalis* subsp. *lactis* strain BB-12	Infants who received probiotics had a higher abundance of *Bifidobacterium* and a reduced presence of *Enterococcus* compared to the placebo group during the supplementation period
Castanet *et al.*^[45]^	Probiotic: *B. animalis* subsp. *lactis* Prebiotic: BMOS	A significant correlation between changes in microbiota composition and the gut maturation marker calprotectin was found. While *B. animalis* subsp. *lactis* increased in the probiotic-supplemented groups, it remained a minor part of the overall fecal *Bifidobacterium* composition. The authors concluded that the prebiotic component of the synbiotic mixture had a more substantial impact on the observed shift in gut microbiota than the probiotic component
Martí *et al.*^[46]^	*L. reuteri* strain DSM 17938	Probiotic supplementation led to greater bacterial diversity and an increased abundance of *L. reuteri* during the first month. At one week, supplementation also resulted in a reduced abundance of *Enterobacteriaceae* and *Staphylococcaceae*. No significant effects were observed at two years
Bargheet *et al.*^[47]^	*B. longum* subsp. *infantis* strain ATCC 15697 and *Lactobacillus acidophilus* strain ATCC 4356	Both microbiota-altering treatments (antibiotics and probiotics) were associated with an increased presence of mobile genetic elements in preterm infants compared to term controls. Thus, antibiotics and probiotics contribute to dynamic changes in the resistome, mobilome, and gut microbiota, which are relevant to infection risk

GM: Gut microbiome; GOS: galactooligosaccharide; BMOS: bovine milk-derived oligosaccharides.

In several studies on probiotic supplementation during pregnancy and infancy, diverse effects on GM composition and immune development have been reported. Rinne *et al.* evaluated the effects of *Lacticaseibacillus rhamnosus* (*L. rhamnosus*) strain GG on infant gut microbiota and immune markers in a study of 96 infants whose mothers received either a probiotic or placebo before delivery, and infants continued the assigned treatment postnatally^[27]^. At 6 months, breastfed infants supplemented with probiotics showed higher counts of *Bifidobacterium* and *Lactobacillus/Enterococcus*. Additionally, probiotic-supplemented infants exhibited increased IgG-secreting cells at 3 months and higher IgM, IgA, and IgG-cell counts at 12 months compared to the placebo group, which suggests that probiotics administered during breastfeeding may positively influence gut immunity. Gueimonde *et al.* examined maternal supplementation with *L. rhamnosus* strain GG from 2-4 weeks before delivery until 3 weeks postpartum in 82 infants^[28]^. At 5 days of age, infants in the probiotic group showed higher colonization of *Bifidobacterium breve* (*B. breve*), but this effect did not persist at 3 weeks. These authors concluded that the transfer and initial establishment of bifidobacteria in neonates result from maternal consumption of *L. rhamnosus* strain GG. Rinne *et al.* studied 132 newborns whose mothers received either probiotics or placebo before and for 6 months postnatally^[29]^. The probiotic treatment did not significantly alter overall gut microbiota composition, but at 6 months, infants in the probiotic group had higher clostridia levels compared to placebo, indicating its role in microbiota succession. Grönlund *et al.* studied the colonization of *Bifidobacterium* species in 61 mother-infant pairs, with mothers receiving probiotics from 30-35 weeks of gestation^[30]^. Only infants of allergic mothers were colonized with *Bifidobacterium adolescentis* (*B. adolescentis*), and their mothers had significantly lower bifidobacterial levels in milk. Consequently, the presence of bacteria in breast milk should be recognized as a key contributor to the development of the intestinal microbiota in infants. Kukkonen *et al.* tested a combination of probiotics and prebiotics in 1,223 pregnant women, showing that probiotic-supplemented infants had higher colonization by lactobacilli and *Propionibacterium* at 3 and 6 months, although the intervention did not reduce allergic disease incidence by age 2 years^[31]^. Building on these findings, the authors posit an inverse correlation between atopic diseases and gut colonization by probiotics. Abrahamsson *et al.* assessed the effects of *Limosilactobacillus reuteri* (*L. reuteri*) supplementation, finding that at 5 days, the probiotic group had significantly higher colonization, but prevalence declined over time^[32]^. However, the probiotic *L. reuteri* was found in breast milk in nearly all infants following oral supplementation during the first year of life, and in some infants who were not treated. Niers *et al.* evaluated a mixture of probiotics administered prenatally to mothers of high-risk children, showing a preventive effect on eczema that persisted until age 2^[33]^. This preventive effect, related to probiotics, appears to be established within the first 3 months of life. Grönlund *et al.* applied two probiotic combinations in 61 mother-infant pairs, finding significant effects on maternal-infant *Bifidobacterium* counts, but no impact on colonization frequencies^[34]^. The authors concluded that maternal colonization by *Bifidobacterium bifidum* (*B. bifidum*) had the most consistent effects on the infant’s bifidobacterial microbiota. Conversely, maternal probiotic treatment had minimal impact on the aforementioned mother-infant association. Grześkowiak *et al.* used two probiotic combinations in 57 mother-infant pairs, showing significant differences in *Lactobacillus*-*Enterococcus* counts, but no major shifts in other bacterial groups^[35]^. Ismail *et al.* found no significant impact of prenatal *L. rhamnosus* strain GG supplementation on infant microbial diversity^[36]^. Probiotic treatment had varying effects on the GM composition of Finnish and German infants, attributable to discrepancies in feeding modes and early commensal microbiota. The authors also reported that maternal administration of *L. rhamnosus* strain GG during the late stages of pregnancy did not modulate diversity in the early infant gut microbiota, despite promoting a beneficial bifidobacteria profile. Pärtty *et al.* reported that 94 preterm infants receiving *L. rhamnosus* strain GG, prebiotics, or placebo did not show significant effects on overall microbial diversity, but the probiotic group had lower *Clostridium histolyticum* levels^[37]^. Furthermore, the provision of early prebiotic and probiotic supplementation was shown to mitigate symptoms related to crying and fussing in preterm infants, suggesting a novel preventive approach for this prevalent disturbance in early life. Enomoto *et al.* showed that probiotic supplementation with *Bifidobacterium longum* (*B. longum*) and *B. breve* resulted in higher Bacteroidota abundance in infants at 4 months, although no differences were observed at 10 months^[38]^. These findings indicate that the administration of bifidobacteria during the prenatal and postnatal periods is effective in preventing allergic diseases. Bisanz *et al.* noted that *L. rhamnosus* supplementation to mothers led to higher *Bifidobacterium* relative abundance in infants at 10-25 days^[39]^. The consumption of Moringa-supplemented probiotic yogurt was shown to enhance *Bifidobacterium* relative abundance and to reduce the presence of *Enterobacteriaceae* in the feces of newborns. However, these effects were not observed in the maternal microbiota across all body sites. The oral and gut microbiota remained stable throughout pregnancy, while the vaginal microbiota exhibited a substantial increase in diversity around birth and in the postpartum period. Rutten *et al.* pointed out that a probiotic mixture administered to 1,099 preterm infants led to a higher prevalence of probiotic species during supplementation, but these differences were not sustained after cessation^[40]^. Perinatal probiotic treatment in children at high risk for atopic disease had minimal effects on GM composition during the supplementation period. No lasting differences were identified by the authors, suggesting that, regardless of intervention or atopic disease status, children follow a common microbiota development trajectory over time, influenced by age, which persists between two and six years of age. Avershina *et al.* administered *L. rhamnosus* strain GG in 48 mother-infant pairs, finding a greater relative abundance of this bacterium at 10 days and 3 months, but no significant differences in microbiota diversity at 12 months or 2 years^[41]^. The authors concluded that the late-colonizing OTUs were acquired at a later stage and not at birth. Korpela *et al.* tested a probiotic mixture and prebiotic in 96 mother-infant pairs, showing that in breastfed infants, probiotics increased *Lactobacillus* and *Bifidobacterium* relative abundance^[42]^. In this regard, in formula-fed infants, *Bifidobacterium* abundance was lower, with other taxa showing increases. The findings of the study demonstrate the efficacy of probiotic supplementation in conjunction with breastfeeding in rectifying adverse disruptions in the composition and function of the infant’s microbiota. These changes may result from antibiotic treatments or cesarean delivery. In turn, Pärnänen *et al.* found no significant impact of two probiotic combinations on antibiotic resistance genes^[43]^. The authors posited that infants inherit their mothers’ legacy of past antibiotic consumption, a phenomenon transmitted genetically. However, the composition of the microbiota remains a significant factor in determining the overall resistance load. Plummer *et al.* studied 1,099 preterm infants, showing higher *Bifidobacterium* and reduced *Enterococcus* in the probiotic group during supplementation^[44]^. The authors identified a correlation between increased *Bifidobacterium* abundance in the immediate postnatal period and a reduced risk of necrotizing enterocolitis in very preterm infants. Furthermore, Castanet *et al.* investigated the effects of different nutrient combinations in infants fed starter formula, finding that prebiotic components had a greater impact on microbiota shifts than probiotics^[45]^. A correlation was noted between alterations in microbiota composition and the gut maturation marker calprotectin. Supplementation with the prebiotic seems to promote a more advanced state of gut maturation, resembling that observed in breastfed infants. Moreover, Martí *et al.* conducted a study in which they administered a *L. reuteri* supplementation to 132 extremely preterm infants, noting increased bacterial diversity but no significant long-term effects^[46]^. Overall, probiotics appeared to have the potential to confer benefits by modulating the composition of the GM during the initial postnatal period (the first month) in infants with extremely low birth weight. Lastly, Bargheet *et al.* tested probiotic effects in preterm infants, showing improved microbiota and resistome similarity to term infants, but both probiotics and antibiotics increased the presence of mobile genetic elements^[47]^. The authors concluded that prolonged hospitalizations, antibiotic use, and probiotic interventions contribute to dynamic alterations in both the resistome and mobilome, which are key characteristics of the gut microbiota central to infection risk.

**Figure 1:**
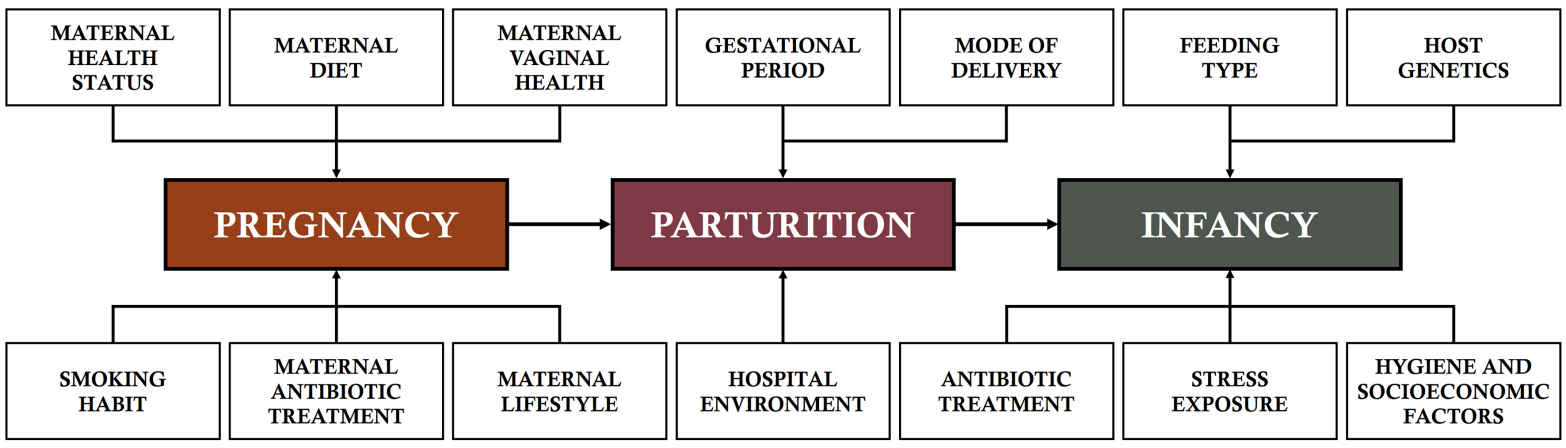
Important factors affecting microbiome abundance and richness at the early stage of life.

The establishment of the GM infant shape is impacted by a variety of external, maternal-related, nutritional, and pharmacological agents^[48-50]^. Following birth, the initial microorganisms that colonize the body of the infant are derived from the maternal microbiota, including sources such as the vagina, skin, mouth, and feces, along with microbes from the immediate environment^[51]^. The predominant bacterial composition of the GM of vaginally delivered newborns is the genera *Bifidobacterium*, *Collinsella*, *Clostridium*, *Lactobacillus*, *Streptococcus*, *Veillonella*, *Bacteroides*, *Parabacteroides*, *Prevotella*, *Sneathia*, *Escherichia*, *Shigella*, and *Akkermansia*^[51-54]^. Alternatively, the GM of cesarean-born infants is primarily composed of *Corynebacterium*, *Propionibacterium*, *Slackia*, *Staphylococcus*, *Streptococcus*, *Veillonella*, *Enterobacter*, and *Haemophilus*^[5,21,55-58]^. Figure 1 presents several important factors affecting microbiome abundance and richness at the early stage of life.

During the first three months of life, breastfeeding as a method of infant nutrition leads to changes in the composition of the GM, resulting in increased levels of the genera *Bifidobacterium*, *Corynebacterium*, *Propionibacterium*, *Sneathia*, *Enterococcus*, *Lactobacillus*, and *Streptococcus*, and decreased levels of *Bacteroides* and *Staphylococcus*^[59-61]^. Nevertheless, formula-feed infants possess a recognizable GM composition, mainly characterized by elevated levels of the bacterial genera *Atopobium*, *Clostridium*, *Enterococcus*, *Granulicatella*, *Lactobacillus*, *Bacteroides*, *Citrobacter*, *Enterobacter*, *Escherichia*, and *Bilophila*^[58,59,62,63]^.

In the course of weaning, the introduction of various solid foods and novel dietary components leads to a rise in microbial α-diversity and pH within the GM^[5]^. Solid foods promote the proliferation of bacteria capable of utilizing a broader spectrum of carbohydrates, synthesizing vitamins, and degrading xenobiotics^[57,64-66]^. Consequently, the dominant members of the infant microbiome undergo a shift, although there is a substantial difference between the GM of infants who have weaned and those who have been breastfed for a continued period. In the initially mentioned group, the predominant genera include *Bifidobacterium*, *Anaerostipes*, *Blautia*, *Clostridium*, *Faecalibacterium*, *Roseburia*, *Ruminococcus*, *Bacteroides*, *Bilophila*, and *Akkermansia*. In contrast, infants who continued breastfeeding for an extended duration exhibit higher abundances of *Collinsella*, *Lactobacillus*, *Megasphaera*, and *Veillonella*^[5,57,67]^. These microbial alterations are linked to enhanced protein intake (associated with members of the family *Lachnospiraceae*), heightened dietary fiber intake (connected to members of the family *Prevotellacea*), and increased mucin generation (from the genus *Akkermansia*)^[60]^. It is estimated that approximately three years are required for the establishment of a mature and functional GM, at which point its composition resembles that of adults^[66,68,69]^. Nevertheless, the structure and composition of the GM are continually and dynamically influenced throughout life by factors such as drug use, dietary patterns, physiological changes, infectious diseases, and lifestyle choices^[66,70-73]^.

## IMPACT OF EARLY-LIFE MICROBIOTA ON LONG-TERM PHYSIOLOGICAL OUTCOMES

The early establishment of microbial communities plays a crucial role in the parallel development of the immune system and the subsequent maturation of the gut and its associated metabolic functions. Therefore, GM dysbiosis may disrupt or alter this programming, resulting in long-term physiological responses and health conditions^[11]^. In this sense, it has been demonstrated that microbial factors influence the activity of chemokine ligand CXCL16, which regulates the concentration of non-variable natural killer T cells in both the colon and lungs. Furthermore, colonizing germ-free mice with a conventional microbiota during the neonatal period protects against this accumulation^[74]^. According to these authors, the early-life microbiota initiates enduring effects, and the lack of such microbial exposure may lead to inflammatory responses later in life that are associated with asthma and IBDs. More recently, a link has been suggested between the disruption of the GM balance and the sustained impacts on immune system disorders^[75]^. This cross-talk occurs through host-microbial interactions in the initial days of life, or via microbial acquisition in gestation, indicating that the risk of disease may be established early in life, including during the prenatal period^[60]^.

### Allergic diseases

Among the immune pathologies associated with the establishment of a specific microbiota, allergies, especially in the form of atopic dermatitis (AD) and subsequently asthma, are probably the result of inadequate GM development and the consequent disturbance of immune homeostasis in the initial year of existence^[76,77]^. In the case of AD or eczema, several classic studies have provided evidence of early shifts in the microbiota of infants who later developed this skin disease^[20,78,79]^. These investigations revealed differences in the microbial composition of infant GM, with an increased abundance of clostridia and *Escherichia coli*, and diminished levels of bifidobacteria and *Faecalibacterium* for the later development of allergic disease^[80,81]^. Further research has shown that a decreased microbial α- and β-diversity of the early-life microbiota and depletion of *Bacteroides* and *Clostridium sensu stricto 1* directly correlate with later development of eczema at one year of age^[82-84]^, and a reduction in eczema severity during the three-month follow-up interval was directly associated with an enhancement in butyrate-producing bacteria, such as *Coprococcus eutactus*^[85,86]^.

Although the development of asthma has been associated with genetic, epigenetic, and environmental factors^[87,88]^, there is a growing recognition of the critical role that the GM plays in the perinatal programming of this condition^[89,90]^. The concept of the “gut-lung axis” illustrates the influence of the GM on lung immune function, both through direct activation of the innate immune response and indirectly via the metabolites generated by gut microbes. The colonization of the intestinal microbes in newborns is pivotal for their overall health, with dysbiosis occurring in the first 100 days and being particularly impactful for the development of hypersensitivity disorders^[91]^. Infants at risk for asthma have significantly reduced relative abundances of the genera *Rothia*, *Faecalibacterium*, *Lachnospira*, and *Veillonella*, and these dissimilarities in bacterial taxa abundance were also associated with distinct amounts of microbial metabolites in feces^[92]^. Additionally, lower gut microbiota diversity in the first month of life has been associated with an increased incidence of asthma in children by age seven^[93]^. Moreover, reduced levels of *Lachnospira* combined with elevated levels of *Clostridium* spp., especially *Clostridioides difficile*, during infancy are positively correlated with a greater risk of asthma development by the age of four or older^[94,95]^.

### Metabolic disorders

The composition and function of the GM have been related to metabolic disorders, such as obesity and obesity-related diseases. Adequate gut barrier function appears to be pivotal for metabolic health^[96]^, but various factors that disrupt this barrier and microbial eubiosis during early life play a critical role in overweight, obesity development, and childhood adiposity later in life. The GM, by increasing energy expenditure, may regulate obesity behavior and peripheral metabolism through the so-called obesogenic microbiota^[97]^. Several studies have indicated that various factors that impact the establishment of the GM during infancy could contribute to the risk of obesity later in life, such as feed, maternal obesity, mode of delivery, intestinal permeability, pathogenic infections, and antibiotic exposure^[98-100]^. Microbiota-related obesity studies in humans have indicated that early microbial profiles may serve as predictors for overweight in children^[101]^. In this sense, it has been reported that overweight in seven-year-old children is associated with increased abundance of members of the phylum Bacillota and decreased abundance of the genus *Bifidobacterium*^[102]^, while *Bacteroides fragilis* levels at 1 month of age were significantly correlated with an increased body mass index in children^[103]^.

In addition, epigenetic shifts linked to microbiota in the early stages of development^[104]^, and also the influence of the GM on brain development, must be considered in the programming of obesity^[105]^. According to some preclinical and clinical studies, the most important factor influencing metabolic diseases is antibiotic therapy in early life^[106]^, which significantly alters the GM^[100,107,108]^. However, one study involving a substantial cohort of over 260,000 subjects reported that childhood obesity was positively associated only with non-treated infections rather than with antibiotic intake during infancy^[109]^. To resolve these conflicting findings, further evidence from human epidemiologic studies is necessary to conclusively determine the causal relationship between antibiotic-driven dysbiosis during early life and subsequent metabolic effects in later life.

### T1D

Dysbiosis of the GM in early infancy has also been linked to various chronic diseases that may emerge later in life. T1D is an autoimmune disease that results from an autoimmune response in which autoreactive T cells partially or completely destroy the beta cells responsible for insulin production within the islets of the pancreas, and it is triggered by genetic and environmental factors^[110,111]^. Accumulating evidence from both preclinical and human studies suggests a role for GM in the onset of this condition^[112,113]^. Microbial studies in T1D have shown a lower microbial diversity and a significant difference in the Bacillota/Bacteriodota phyla ratio, and also diminished levels of the butyrate producer *Faecalibacterium prausnitzii* in children with diabetes^[114,115]^. Interestingly, several studies have suggested that early-life gut colonization may influence the course of T1D development and is also involved in the pathophysiology of this disease^[116]^. In the DIABIMMUNE study, children from different geographical contexts, specifically Estonia, Finland, and Russia, who had an HLA predisposition to autoimmune diseases, were examined^[117,118]^. The children who developed T1D showed a reduction in α-diversity and elevated levels of *Blautia*, *Rikenellaceae*, *Ruminococcus*, and *Streptococcus*, while *Coprococcus eutactus* and *Dialister invisus* were absent. A separate study involving 33 infants at risk for T1D revealed a reduction in bacterial diversity and an increase in pro-inflammatory bacterial species, including *Ruminococcus gnavus* and *Streptococcus infantarius*^[119]^. In addition, the researchers observed heightened levels of human β-defensin 2, an antimicrobial molecule synthesized during inflammation, in those children who went on to develop T1D.

### IBDs

IBDs are hyperimmune, multifactorial diseases that include Crohn’s disease and ulcerative colitis. Both disorders are related to inflammation and changes in the GM (e.g., decreased microbial diversity and lower abundance of *Roseburia*) and have a strong genetic basis^[120]^. These diseases may first appear in childhood and adolescence and have a lifelong chronic, relapsing course^[121]^. Several factors have been reported to be associated with the development of IBDs in childhood, such as exposure to antibiotics and cigarette smoke during fetal life, and also breastfeeding^[122,123]^. Although infants born to mothers with IBDs showed an increased abundance of members of the phylum Pseudomonadota and a decreased abundance of bifidobacteria during the first 3 months of life^[124]^, it is still not clear whether these associations are causal or interrelated, as the persistent inflammation of IBDs may affect the GM rather than the dysbiosis that causes IBDs^[125]^.

### ACVDs

Specific conditions in infancy, including preterm birth, malnutrition, or the colonization of the GM, may increase the risk for an individual to develop ACVDs later in life^[126]^. Malnutrition and changes in the GM composition are closely related, as a decline in commensal gut bacteria, such as *Bifidobacterium*, can result in poor digestion, and in turn, decreased use of dietary carbohydrates and diminished vitamin synthesis may contribute to malnutrition^[126,127]^. In addition, elevated levels of Pseudomonadota in preterm infants have been identified in certain adults with ACVDs^[128]^. Phylum Pseudomonadota contains several pathobionts that reduce nutrient absorption, potentially causing epithelial damage and promoting inflammation, which in turn compromises intestinal barrier permeability^[121]^. This situation can escalate systemic inflammation, a key factor in the development of ACVDs^[129,130]^.

ACVDs are characterized by chronic inflammation in which lipids are retained within the arterial wall, leading vascular smooth muscle cells to form a collagenous, fibrous cap that becomes infiltrated by immune cells, such as mast cells, T cells, and macrophages^[131]^. This atherosclerotic process, a primary contributor to ACVDs, begins early in life and is linked to a broad range of risk factors, such as diabetes, hypertension, persistent low-grade inflammation, and GM imbalances^[132]^. Therefore, the impacts of gut metabolites and gut dysbiosis underscore the influence of the GM on ACVDs by promoting inflammation and altering cholesterol metabolism. Bacterial presence has been detected within atherosclerotic plaques^[133]^, contributing to the origination of atherogenesis by stimulating platelet aggregation and thrombus formation, or by acting via their structural components like LPS, to activate an inflammatory cascade by heightening the expression of IL-1β^[134]^. Diverse studies have examined the indirect impact of the GM on ACVD development via its metabolites [short-chain fatty acids (SCFAs), trimethylamine, trimethylamine-N-oxide (TMAO), and bile acids (BAs)], which regulate host systemic inflammation, activate the innate immune system, and shape the adaptive immune response^[121,135,136]^. These microbial metabolites can function as signaling molecules, binding to specialized receptors on remote organs or influencing endocrine pathways through indirect interactions with other endocrine molecules^[134]^.

## IMPACT OF ELS ON THE GM

ELS encompasses a range of adverse experiences occurring before the age of 18, including forms of abuse (psychological, physical, or sexual), neglect (both emotional and physical), persistent family dysfunction, and socioeconomic struggles^[137]^. In addition, ELS is a predictor of adult depression^[138]^, and it is related to the magnitude of depressive symptoms and the duration of the depressive trajectory^[139]^. Several studies have shown that ELS provokes GM dysbiosis^[140]^, and that the GM exerts a pivotal role in the development of depression through the gut-brain axis communication^[141,142]^. The interplay between GM and depression constitutes a reciprocal process; depressed patients exhibit altered GM composition^[143]^, and the transplantation of GM from individuals with depression can result in anxiety and depressive behaviors in receptor rodents^[144]^.

The human GM composition shows marked differences between individuals with depression and healthy controls, but there are controversial results across studies^[145,146]^. At the phylum level, findings from most studies indicate that individuals with depression exhibit a significantly higher relative abundance of Actinomycetota compared to controls^[147-152]^. At the family level, the most abundant taxa in depressed patients include *Bifidobacteriaceae*, *Enterobacteriaceae*, and *Lachnospiraceae*^[147-149,152,153]^. At the genus level, the most abundant bacteria are *Alistipes*, *Bacteroides*, *Bifidobacterium*, *Blautia*, *Clostridium*, *Eggerthella*, *Holdemania*, *Oscillibacter*, *Parabacteroides*, and *Streptococcus*^[143,148-156]^. On the other hand, members of the phylum Bacteroidota and genus *Faecalibacterium* seem to be inversely related to depression^[147,148,150-153]^. More recently, Kraaij *et al.* performed a cross-sectional study involving 1,784 ten-year-old children from the Netherlands to define the relationships between the GM and mental health issues in children^[157]^. Although lower gut microbial diversity and richness were associated with internalizing problems and anxious/depressed behavior issues, these associations were not significant. These authors did not find definitive evidence linking GM diversity, taxonomic features or functions, and mental health conditions in the pediatric cohort. However, they noted suggestive findings indicating a reduction in the genera that have previously been related to psychiatric disorders, including *Anaerotruncus*, *Hungatella*, and *Oscillospiraceae*. No associations were found between ELS and the GM, although socioeconomic stress was the only ELS domain associated with lower α- and β-microbial diversity^[158]^. Table 2 shows several studies examining changes in the GM composition of patients with depression.

**Table 2:** Alterations in the GM composition observed in depressed patients

**Study**	**Participants**	**Sequencing methods**	**Increased**	**Decreased**
Naseribafrouei *et al.*^[155]^	*N* = 37 depressed patients (mean age 49.2 years) and *N* = 18 HCs (mean age 49.2 years)	16S rRNA	Order bacteroidales Genera: *Alistipes* and *Oscillibacter*	Family: *Lachnospiraceae*
Jiang *et al.*^[149]^	*N* = 46 depressed patients and *N* = 30 HCs Age rank: 18-40 years	Pyrosequencing	Phyla: Actinomycetota, Bacteroidota, and Pseudomonadota Family: *Enterobacteriaceae* Genus: *Alistipes*	Phylum: Bacillota Genus: *Faecalibacterium*
Zheng *et al.*^[152]^	*N* = 165 subjects with MDD and *N* = 217 HCs	16S rRNA	Phylum: Actinomycetota Families: *Actinomycetaceae*, *Coriobacteriaceae*, *Enterobacteriaceae*, *Lachnospiraceae*, *Lactobacteriaceae*, *Ruminococcaceae*, and *Streptococcaceae* Genera: *Anaerostipes*, *Blautia*, *Clostridiales incertae sedis XI*, *Dorea*, *Erysipelotrichaceae incertae sedis*, and *Parvimonas*	Phylum: Bacteroidota Families: *Acidaminococcaceae*, *Bacteroidaceae*, *Rikenellaceae*, *Sutterellaceae*, and *Veillonellaceae* Genera: *Alistipes*, *Clostridium XlVa*, *Coprococcus*, *Faecalibacterium*, *Lachnospiracea incertae sedis*, *Megamonas*, *Phascolarctobacterium*, and *Roseburia*
Chen *et al.*^[147]^	*N* = 10 MDD patients (age, 18-56 years) and *N* = 10 HCs (age, 24-65 years)	Metaproteomics	Phyla: Actinomycetota, and Bacillota Families: *Actinomycetaceae*, *Bifidobacteriaceae*, *Clostridiaceae*, *Erysipelotrichaceae*, *Lachnospiraceae*, *Nocardiaceae*, *Porphyromonadaceae*, *Ruminococcaceae*, and *Streptomycetaceae*	Phyla: Bacteroidota and Pseudomonadota Families: *Chitinophagaceae*, *Enterobacteriaceae*, *Marinilabiliaceae*, *Oscillospiraceae*, *Prevotellaceae*, *Rikenellaceae*, and *Sutterellaceae* Genus: *Faecalibacterium*
Chung *et al.*^[148]^	*N* = 36 MDD patients and *N* = 37 HCs Age rank: 20-65 years	16S rRNA	Phylum: Actinomycetota Families: *Bifidobacteriaceae*, *Lachnospiraceae Peptostreptococcaceae*, *Porphyromonadaceae*, and *Streptococcaceae* Genera: *Adlercreutzia*, *Bifidobacterium*, *Clostridium cluster X I*, *Eggerthella*, *Holdemania*, *Parabacteroides*, *Ruminococcus*, and *Streptococcus*	Phyla: Bacteroidota and Pseudomonadota Families: *Alcaligenaceae* and *Prevotellaceae* Genera: *Megamonas*, *Prevotella*, and *Sutterella*
Rong *et al.*^[151]^	*N* = 31 depressed subjects (mean age 41.6 years), and *N* = 30 HCs (mean age 39.5 years)	Shotgun metagenomics	Phyla: Actinomycetota, and Bacillota Genera: *Bacteroides*, *Bifidobacterium*, *Clostridium*, *Oscillibacter*, and *Streptococcus*	Phylum: Bacteroidota
Yang *et al.*^[143]^	*N* = 156 MDD patients and *N* = 155 HCs Age rank: 18-65 years	Metagenomic	Genus: *Bacteroides*	Genera: *Blautia* and *Eubacterium*
Zheng *et al.*^[146]^	*N* = 165 MDD patients (mean age 26.5 years) and *N* = 217 HCs (mean age 26.8 years)	16S rRNA	Families: *Bacteroidaceae* and *Bifidobacteriaceae*	Family: *Enterobacteriaceae*
Lai *et al.*^[150]^	*N* = 26 depressed patients and *N* = 29 HCs	Shotgun metagenomic	Phylum: Actinomycetota Genera: *Atopobium*, *Bifidobacterium*, *Coriobacterium*, *Eggerthella*, *Olsenella*, *Rothia* and *Slackia*	Phylum: Bacteroidota
Stevens *et al.*^[153]^	*N* = 20 depressed patients and *N* = 20 HCs (mean age 34 years)	16S rRNA	Families: *Acidaminococcaceae*, *Coriobacteriaceae*, and *Enterobacteriaceae* Genera: *Alistipes*, *Blautia*, *Flavonifractor*, *Holdemania*, *Oscillibacter*, *Parabacteroides*, *Phascolarctobacterium* and *Roseburia*	Family: *Lachnospiraceae* Genera: *Bacteroides*, *Faecalibacterium*, and *Ruminococcus*
Mayneris-Perxachs *et al.*^[154]^	*N* = 25 depressed patients, *N* = 25 MDD and *N* = 44 HCs	Shotgun metagenomic	Genera: *Acidaminococcus* and *Parabacteroides*	Family: *Lachnospiraceae* Genus: *Bifidobacterium*

GM: Gut microbiome; HCs: healthy controls; MDD: major depressive disorder.

Retrospective studies have shown a consistent association between ELS and cognitive decline in adulthood, linked to systemic inflammation^[159,160]^. ELS has also been related to neurological deficits in executive function, memory capacity, and processing speed^[161-163]^, which are associated with significant changes in the hippocampus and the prefrontal cortex^[164]^, thereby affecting the hypothalamic-pituitary-adrenal (HPA) axis and the neuroendocrine system, both implicated in stress regulation due to the release of cortisol^[165]^. Cortisol influences a range of cognitive and physiological processes, including immunity, inflammation, and neuroplasticity^[166]^. Additionally, individuals subjected to ELS frequently present psychiatric comorbidity with multiple behavioral consequences^[167-169]^. Moreover, individuals who have experienced childhood and adolescent psychological trauma possess significant difficulties in regulating their emotions, limitations regarding their social interactions, reduced capacity to concentrate, and persistent psychological distress that persists into adulthood^[170]^.

Chronic stress, in combination with GM dysbiosis, has been shown to disrupt SCFA metabolism and exacerbate dysfunction in the microbiota-gut-brain axis in individuals with depression. SCFAs exhibit neuroprotective effects and are involved in pathological processes linked to the onset and progression of depression, such as neuroinflammation, neuroendocrine fluctuations, chronic cerebral hypoperfusion, and epigenetic modifications^[171]^.

SCFAs present in the systemic circulation are capable of crossing the blood-brain barrier (BBB), thereby modulating the transfer of nutrients and molecules that are instrumental in preserving the integrity of the BBB. This process exerts a direct influence on brain development and the maintenance of central nervous system (CNS) homeostasis^[172]^. Furthermore, SCFAs have been shown to regulate a multitude of fundamental behavioral and neurological processes by modulating the HPA axis, the immune system, and tryptophan metabolism, as well as by contributing to the synthesis of various metabolites, including neurotransmitters with neuroactive properties^[173]^.

Within the microbiota-gut-brain axis, SCFAs play a pivotal role in the synthesis and release of peripheral neurotransmitters, such as acetylcholine and serotonin (5-HT)^[174]^. However, the permeability of the BBB can limit the access of these neurotransmitters into the brain, potentially hindering their ability to directly affect CNS function. Although peripheral blood 5-HT has been shown to regulate gastrointestinal motility and excretion, it may also constitute a potential indirect mechanism by which cognitive, emotional, and behavioral responses are influenced via neuroendocrine pathways or vagal afferents^[175]^. In addition, studies have demonstrated that, upon crossing the BBB, SCFAs modulate neurotransmitter levels within the CNS^[176]^.

## DISCUSSION AND FINAL REMARKS

The present review aimed to summarize the impact of early-life GM development and dysbiosis on long-term health, focusing on its role in physiological and mental health. Reinforcing key findings, a large body of recent studies have explored this topic, primarily focusing on psychosocial factors^[177,178]^, acute stress^[179-182]^, mental disorders^[183]^, as well as physiological, metabolic, and immune processes^[184]^. However, much still remains to be elucidated regarding the underlying mechanisms and long-term effects of early-life GM alterations.

Emerging evidence suggests that early-life GM plays a critical role in shaping long-term health, with disruptions during key developmental stages contributing to various chronic conditions. Alterations in GM composition, driven by factors such as maternal stress, early nutrition, and perinatal antibiotics, can have lasting effects on immune and metabolic processes. Moreover, exposure to prenatal and postnatal adversities has been linked to altered GM profiles in children, with specific microbial taxa associated with adversity exposure. These alterations are reflected in the child’s socioemotional functioning, supporting the idea that intergenerational transmission of adversity may affect mental health through changes in GM function^[177]^. Moreover, variations in the GM, along with inflammatory markers, may represent mechanistic pathways for the observed health outcomes. For instance, GM characteristics could predict social disadvantage and psychosocial stress, highlighting microbial imbalances as mediators of early adversity effects^[178]^. This evidence suggests that ELS can induce microbial changes predisposing individuals to conditions like asthma and diabetes.

ELS has also been linked to later-life health issues, including inflammatory diseases and cardio-metabolic disorders^[121,181]^. In addition, ELS-induced GM dysbiosis plays a particularly crucial role in depression through gut-brain axis communication^[142,183]^. Inadequate or insufficient microbial exposure in early life can lead to inflammatory responses associated with several conditions, such as allergies, obesity, T1D, and cardiovascular diseases^[121,184-186]^. Furthermore, childhood trauma has been shown to negatively impact stress recovery, with heart rate indices indicating impaired recovery, further emphasizing the long-term effects of ELS on health. These findings point to enduring impacts on both physical and psychological well-being in adulthood^[179]^. Thus, understanding the mechanisms behind early-life GM dysbiosis is essential for identifying potential interventions to mitigate the risk of chronic diseases.

Maternal stress is another key factor influencing both maternal and infant microbiota. Prenatal and postnatal stress can lead to volatile shifts in infant GM that are specific to certain developmental stages^[180]^, indicating a complex relationship between stress and microbiome development, as well as potentially exacerbating the risk of chronic diseases in offspring. Early exposure to maternal stress may predispose individuals to conditions like obesity, cardiovascular diseases, and neurodevelopmental disorders, with a disrupted GM playing a central role^[181]^. Furthermore, stress-related changes in the microbiome may involve epigenetic modifications that adapt the gut-brain axis to stress^[182]^.

Although several studies have reported a connection between ELS and the GM during prenatal and postnatal periods, a unified microbiome signature linked to either prenatal or postnatal stress has not yet been completely established^[177,178,180]^. This variability in findings is likely attributable to a range of methodological differences, including variations in experimental designs, age groups, geographical locations, ethnic backgrounds, assessment tools, timing of sample collection, analysis techniques, sample sizes, and the nature of stressors examined. In addition, differences in microbial composition across regions or populations, as well as the source of the samples (e.g., human *vs.* animal models, or hospital *vs.* community-based samples), can contribute to observed inconsistencies. These factors may impact the generalizability and comparability of results. Further research employing consistent stressors, validated stress metrics, and high-resolution microbiome analyses is essential to establish clear connections between stress and the human GM^[187,188]^.

Mulder *et al.* found that specific domains of ELS, such as socioeconomic stress, presented limited evidence of association with the GM, suggesting that other factors may also be implicated^[158]^. However, the limited number of longitudinal studies and controlled intervention trials on this topic makes it difficult to establish a clear causal relationship. Consequently, future research is necessary to establish causality and determine whether various modifiable factors might be effectively targeted to improve gut health, particularly in children facing heightened contextual stress or adverse conditions. Understanding the mechanisms by which these factors influence the GM is crucial, as it could facilitate the development of customized interventions that mitigate the adverse effects of ELS.

Nutrition has been shown to play a crucial role in shaping microbial composition^[70]^, making a diet rich in essential nutrients, particularly balanced plant-based patterns, ideal for supporting microbial diversity. For example, the adoption of a vegetarian diet, rich in indigestible fibers, facilitates fiber fermentation and alters the intestinal microbial ecosystem, leading to the production of metabolites such as SCFAs and other postbiotics. These metabolites exert beneficial effects on the intestinal immune system, the integrity of the BBB, energy substrate supply, and defenses against microbial pathogens^[189]^.

The modulation of the GM and its metabolites through the administration of psychobiotics also seems to be a very promising approach for treating CNS alterations resulting from ELS^[190]^. Psychobiotics, including probiotics, synbiotics, and postbiotics, provide mental health benefits by modulating the GM, which in turn influences the regulation of stress, anxiety, and depression symptoms^[191]^. With respect to ELS, few studies have investigated the use of probiotics to mitigate its effects on mental health and CNS function, through the ability of probiotics to synthesize neuroactive compounds such as gamma-aminobutyric acid, serotonin, dopamine, norepinephrine, and acetylcholine^[192]^. In addition, Borrego-Ruiz and Borrego reviewed the application of FMT in various neurological and mental health disorders, highlighting overall positive outcomes^[193]^. However, the broader clinical implementation of this procedure is limited by multiple factors, including the time and route of administration, the high cost of treatment, and concerns regarding its safety, tolerability, efficacy, and potential side effects.

Within the context of Nutritional Psychiatry, the combined supplementation of psychobiotics and nutraceuticals may offer a synergistic strategy for treating certain mental health conditions. However, significant gaps remain between epidemiological findings and clinical evidence regarding the role of diet-related factors in managing mental disorders. Future research should focus on exploring the mechanistic pathways that involve the GM and its interaction with the CNS^[194]^. Translating microbiota-related insights into clinical practice presents considerable challenges, such as the inherent complexity of individual microbiomes and the difficulty in establishing causal relationships between dietary interventions and clinical outcomes. While dietary interventions can serve as a supportive measure, they should not be viewed as a cure for severe mental illnesses. Instead, they should be considered part of a broader, comprehensive treatment plan that includes approaches with more robust clinical validation^[195]^.

Addressing psychosocial factors, such as the availability of mental health resources and supportive environments, can further help mitigate the impact of stressors on gut integrity and overall health. In this respect, interventions should not only focus on individual-level approaches but also on transforming the prevailing adverse social dynamics. As individuals progress through life, several potentially disturbing and distressing events, such as the emotional experience of humiliation due to bullying victimization, can lead children and adolescents to severely negative health and behavioral outcomes^[196]^. Therefore, a multidisciplinary approach aimed at addressing the factors that can induce ELS is essential for promoting optimal development and overall well-being for all individuals, including initiatives directed at advancing the understanding of the influence of the microbiome and the potential interventions derived from it, which to date appear to show promising results.

Future research should focus on investigating the causal relationship between microbiota and stress using more rigorous research approaches. Specific experimental designs, such as longitudinal studies and randomized controlled trials, are essential for establishing stronger evidence of causality. Furthermore, advanced analytical techniques, including multivariate analysis, should be employed to better understand the complex interactions involved. It is also crucial to address potential confounding variables, including diet, lifestyle factors, and other environmental influences, to ensure the validity and accuracy of the findings. Taking these factors into account will enhance the ability of future studies to generate conclusive insights into the mechanisms linking the GM to ELS-related health outcomes.

Although substantial progress has been made in understanding the role of the GM in early development, there remains a need for further studies to establish clear causal relationships between GM alterations and long-term health outcomes. Future research should focus on refining our understanding of the temporal dynamics of GM dysbiosis, particularly in the context of ELS, and explore potential therapeutic strategies to restore microbial balance and improve long-term health.

## DECLARATIONS

### Copyright

© The Author(s) 2025.
